# Stress hormones and posttraumatic stress symptoms following paediatric critical illness: an exploratory study

**DOI:** 10.1007/s00787-016-0933-3

**Published:** 2016-12-19

**Authors:** Lorraine C. Als, Maria D. Picouto, Kieran J. O’Donnell, Simon Nadel, Mehrengise Cooper, Christine M. Pierce, Tami Kramer, Vivette A. S. Glover, M. Elena Garralda

**Affiliations:** 1 Department of Medicine, Centre for Mental Health, Imperial College London, Hammersmith Hospital Campus, 7th Floor Commonwealth Building, Du Cane Road, London, W12 0NN UK; 2 Coslada Mental Health Service, Department of Child and Adolescent Psychiatry and Psychology, Hospital Infantil Universitario del Niño Jesús, Madrid, Spain; 3 Douglas Hospital Research Centre, Montreal, Canada; 4 Department of Paediatric Intensive Care, St Mary’s Hospital, Imperial College Healthcare NHS Trust, London, UK; 5 Department of Paediatric Intensive Care, Great Ormond Street Hospital for Children NHS Trust, London, UK; 6 Institute of Reproductive and Developmental Biology, Imperial College London, London, UK

**Keywords:** Salivary cortisol, Critical illness, Psychological sequelae, Post-traumatic stress disorder

## Abstract

In this exploratory case–control study, we investigated basal cortisol regulation in 5–16-year-old children, 3–6 months following PICU (paediatric intensive care) admission. This was nested within a study of child psychological and cognitive function; 47 children were assessed alongside 56 healthy controls. Saliva samples were collected three times per day (immediately after waking, waking +30 min, and waking +12 h) over two consecutive weekdays. In addition, data on posttraumatic stress symptoms were ascertained from 33 PICU admitted children using the Impact of Events Scale-8 (IES-8). Primary analysis revealed no significant differences in basal cortisol concentrations between PICU discharged children and healthy controls (*p* > 0.05). Secondary analysis in the PICU group identified a significant positive association between posttraumatic stress symptoms and evening (waking +12 h) cortisol concentrations (*p* = 0.004). However, when subject to multivariate analysis, evening cortisol was a modest independent predictor of IES-8 scores, relative to the presence of septic illness and poor pre-morbid health. We conclude that paediatric critical illness does not appear to result in marked perturbations to basal cortisol at 3–6 month following discharge. There was evidence of a link between evening cortisol and symptoms of PTSD, but this was not a robust effect and requires further elucidation.

**Electronic supplementary material:**

The online version of this article (doi:10.1007/s00787-016-0933-3) contains supplementary material, which is available to authorized users.

## Introduction

Although improvements in medical care have led to reduced mortality rates following paediatric critical illness, psychosocial sequelae have been demonstrated [1]. We have reported post-traumatic stress symptoms being present in over a third of children following PICU admission, as well as an elevated risk for psychiatric disorder [2].

Little is known of the mechanisms underlying these after-effects, but they are likely to reflect a complex interaction of biological and psychosocial factors. Whilst PICU admission represents a major stress for families [3], critical illness also leads to a cascade of physiological changes. In particular, the activation of the hypothalamic–pituitary–adrenal (HPA) axis brings about changes necessary for an adequate stress response. High cortisol levels have been reported during critical illness in both adults and children, although there is considerable individual variation [4–6]. In children with septic shock and meningococcal disease, both high and low cortisol levels have been associated with increased illness severity and poor physical outcomes [6–9]. Inadequate cortisol levels in the face of severe physiological stress and associations between cortisol and illness severity have also been reported in other critical illnesses [9, 10].

Amongst child survivors of critical illness, cortisol concentrations tend to normalise within 48 h of admission, but the usual circadian rhythms are not immediately restored [6]. High cortisol levels can persist on hospital discharge from intensive care [4].

Activation of the HPA axis is necessary for everyday functioning, but excessive acute stimulation, such as that experienced during critical illness may play a role in the development of subsequent psychological problems. HPA axis dysregulation has been connected with adverse childhood experiences involving maltreatment and with a number of paediatric psychological problems including anxiety and post-traumatic stress disorder (PTSD) [11–16]. In children with traumatic injuries, positive associations have been found between elevated levels of cortisol on admission and PTSD symptoms 6 weeks and 6 months later [17, 18]. It is, therefore, possible that the psychological sequelae of paediatric critical illness are linked to ongoing anomalies in the regulation of the HPA axis.

A commonly used method for assessing HPA axis activity is the measurement of diurnal basal salivary cortisol levels. In the general child population these are highest on waking and are followed by brief early morning reactivity, the so-called cortisol awakening response (CAR), thought to be under genetic influence [11, 19]. Cortisol levels then decrease during the day and are at their lowest in the evening, when they are thought to be related to environmental influences [11, 19, 20].

Whether critical illness affects the subsequent regulation of the HPA axis and whether this is associated with the development of psychological after-effects has not previously been studied in a child population.

In this exploratory study, we compared diurnal variation in basal salivary cortisol in children 3–6 months following discharge from intensive care and in healthy controls, a point in time when most children would be clinically recovered but psychological sequelae already become apparent and could be linked to residual anomalies in cortisol circadian levels. We also examined associations of cortisol with PTSD symptoms in the PICU admitted children. We hypothesised altered diurnal patterns of cortisol in the PICU group in comparison to controls. Furthermore, we postulated that regulatory anomalies would be linked to psychological sequelae in the PICU group.

## Methods

### Participants

The study was nested within a cohort study of psychological and cognitive sequelae in children aged 5–16 years old admitted to intensive care with meningo-encephalitis, sepsis, and other critical illnesses [2, 21].

#### Patient enrolment

Patients included in the nested study were admitted to the PICUs at St Mary’s Hospital and Great Ormond Street Hospital, London UK, May 2008 to March 2010 and followed up 3–6 months after discharge. Since the main study aimed to ascertain whether PICU admission, especially for infectious illness, was likely to impact psychological and cognitive sequelae, we excluded children with a medical history suggesting prior psychiatric, neurological, or developmental disorders and where a psychiatric problem (e.g., deliberate self-harm) or neurological injury (e.g., traumatic brain injury) was the cause of the admission.

#### Community-based controls

Healthy controls with no previous history of PICU admission were recruited from the family and/or friends of patients admitted to PICU as well as local mainstream schools.

### Procedure

#### Collection of cortisol

The measurement of salivary cortisol levels has proved an acceptable, non-invasive, reliable and valid biological marker of HPA activity, both in general populations [22] and during critical illness [23, 24]. Study parents were given a sample collection pack at the 3- to 6-month follow-up appointment containing standardised instructions, six sampling devices called “salivettes” (Sarstedt Inc., Rommelsdorf, Germany), a record sheet and a stamped addressed envelope.

To ensure compliance to the sampling protocol, parents collected the samples, three per day (immediately after waking, waking +30 min, and waking +12 h), over two consecutive weekdays. Instructions indicated that it was important for the child not to eat, drink, or clean their teeth in the 30 min preceding sample collection. The parent was instructed to place or hold (if the child was under 11 years old) the salivette in the child’s mouth for 2 min or until soggy. The specimen was then placed inside a plastic tube, kept in their home fridge until all of the samples had been collected and posted back to the main research site. Research has shown that unfrozen samples remain stable at room temperature for 5–7 days [25]. Upon receipt, the saliva specimens were stored in a −20 °C freezer and transferred to the lab for assay in insulated cold packs in order to keep the thaw cycle to a minimum [26].

On the day of assay, samples were thawed for 30 min and centrifuged for 15 min at 1200*g*. Salivary cortisol was measured using a commercially available enzyme immunoassay (Salimetrics, UK). This competitive binding assay is based on the principle that cortisol in saliva competes with cortisol linked to horseradish peroxide for antibody binding sites. The amount of colour change observed at 450 nm is inversely proportional to the concentration of endogenous cortisol present in each saliva sample. The inter- and intra-assay coefficients of variance were 7.9 and 8.9%, respectively.

Samples that were insufficient for the laboratory analysis or were outliers (e.g., above the detectable limits of the salivary assays or >4 SD above the mean) were excluded from the analyses. Participants with three viable samples (one for each time point) were included in the analysis.

### Questionnaires

#### Socio-demographics and pre-morbid characteristics

A standardised questionnaire gathered information on socio-demographic variables and pre-morbid characteristics such as medical health history (i.e., chronic medical conditions and health problems in the six months prior to PICU admission) and/or emotional or behavioural problems in the child.

#### Clinical indices

Clinical PICU data were obtained from medical records, including length of stay in PICU and hospital, illness severity (Paediatric Illness Mortality score or PIM2 [27]), admission and illness type.

#### Posttraumatic stress symptoms

Child post-traumatic stress symptoms were measured with the eight-item Impact of Events Scale [28] a self-report questionnaire validated for use in children over 8 years. This assesses the presence of symptoms of PTSD, including intrusive re-experiencing of the trauma and avoidance of trauma-related stimuli. The questionnaire asked specifically about the frequency of PTSD symptoms experienced in relation to the PICU admission and occurring in the previous seven days. Items are rated on a 4-point scale (total scores 0–24, a score ≥17 indicating caseness/risk for PTSD) [29]. As the PICU group were all exposed to the stress of critical illness and PICU admission, we analysed post-traumatic stress responses in children 8 years and over in this group only.

### Statistical analysis

Analyses were carried out using the IBM SPSS Statistics for Windows, Version 21.0 (IBM Corp., Armonk, NY, USA). Analyses were two-tailed and significance was set at *p* < 0.05. Due to the small sample size, analysis split by admission type was not feasible. Thus, data are presented for the PICU group as a whole.

The participant characteristics were analysed with nonparametric methods (Mann–Whitney *U* test for continuous and Chi-square test for categorical data). Data are expressed as median (interquartile range [IQR]) and frequency (percent). Bivariate correlations (Spearman’s Rho) were used to assess the possible effects on the cortisol data of possible confounders such as prior chronic illness, use of steroid medication whilst in PICU or taking medications at the time of the cortisol data collection.

Cortisol concentrations were assessed for normality using histograms and Kolmogorov–Smirnov tests. Distributions were skewed and thus log transformation was applied. Cortisol concentrations from sampling days 1 and 2 were significantly correlated (*p* < 0.05), and we therefore used the averaged value of samples for each time-point. Data are expressed as mean (95% CI). We also calculated the cortisol awaking response (CAR), defined as the rise in cortisol within the first 30 min after waking [30] and as a percentage increase over waking levels.

Daytime profiles of cortisol secretion can be analysed by modelling the cortisol variables on a repeated measures multivariate analysis of variance (ANCOVA) or by more indirect variables such as area under the curve (AUC) [19]. For this exploratory study we chose the ANCOVA as being more directly related to the reported data, and as taking into account both within- and between subject effects. Primary analyses involved group comparisons of basal cortisol concentrations across the three time-points using a mixed ANCOVA, with group (PICU vs. healthy controls) as the between-subjects factor, and time (waking, waking +30 min, waking +12 h) as the within-subjects factor. A one-way ANCOVA of the CAR was also conducted. Gender and age were specified as covariates. The role of gender and age (or adolescence, more specifically) has previously been identified as potential confounding variables in relation to basal cortisol secretion across the day [31, 32]. When Mauchly’s test of sphericity was significant, the Greenhouse-Geisser correction was applied to the degrees of freedom.

Secondary analyses were used to explore potential risk factors that could contribute to PTSD symptomatology, with particular interest in basal cortisol concentrations. All variables were first evaluated by univariate linear regression to identify those that were associated with posttraumatic stress symptom scores. The following variables were assessed: (1) vulnerability factors (age, gender, ethnicity, socioeconomic status, family composition (whether intact or not), and pre-existing health problems), (2) critical illness factors (length of stay in PICU, length of stay in hospital, illness severity, type of admission, illness type), and (3) basal cortisol concentrations (waking, waking +30 min, waking +12 h, and CAR). Any variables with a p value less than 0.1 on the univariate analysis were entered into a multivariate regression analysis. Hierarchical (blockwise entry) was used, with vulnerability factors entered in the first block, critical illness factors entered in the second block, and basal cortisol concentrations in the third block. In order to test the assumption of normality of standardised residuals, histograms and the Kolmogorov–Smirnov test were used. All residuals were deemed normally distributed.

## Results

Due to the delayed start of the nested study, not all participants taking part in the main study were approached to take part in the sub-study. Table 1 outlines participation rates. There were 67 insufficient and 17 outlying cortisol samples, and as a result 16 participants were excluded (six PICU patients and ten controls).

**Table 1:** Recruitment into the study

	PICU cohort	Healthy controls
Invited to take part in study	175	Convenience sample
Study participants	88	93
Invited to provide saliva samples	69	83
Consented to participate	67	78
Provided saliva specimens	53	66
Valid salivary data	47	56

Baseline characteristics are detailed in Table 2. Children in the PICU and healthy control groups were similar on most variables. However, the presence of a chronic medical condition was more common in the PICU group (*p* < 0.01). The median age on admission in the PICU group was 10 years (IQR 6–14); children spent a median of 3 days (1–5) on PICU and 11 days (6–21) in hospital; the mean Paediatric Illness Mortality (PIM2) score was 0.06 (0.04–0.15); 11 (23.4%) were admitted with meningo-encephalitis, 17 (36.2%) with sepsis and 19 (40.4%) with another critical illness.

**Table 2:** Baseline characteristics of paediatric intensive care unit group and healthy control group

	PICU cohort (*n* = 47)	Healthy controls (*n* = 56)
Age (years)	10.00 (7.00, 14.00)	11.00 (8.25, 13.00)
Gender		
Male	31 (66%)	26 (46%)
Female	16 (34%)	30 (54%)
Socio-economic status^a^		
Level I	18 (46%)	28 (56%)
Level II	10 (26%)	13 (26%)
Level III	11 (28%)	9 (18%)
Not assigned	2 (5%)	3 (6%)
Ethnicity		
White	26 (55%)	27 (48%)
Other	21 (45%)	29 (52%)
Complications during pregnancy^b^	12 (27%)	14 (25%)
Neonatal special care^b^	6 (13%)	3 (5%)
Family composition^b^		
Intact home	30 (67%)	45 (82%)
Chronic medical condition^c,^*	21 (45%)	9 (17%)
Past general health^b^		
Intermediate/poor	8 (18%)	5 (9%)
Prior emotional/behavioural difficulties^d^	4 (9%)	7 (13%)

Data are median (inter-quartile range) or frequency (percent)

The “not assigned” category included parents that were unemployed, students, or retired

^a^Data available for *n* = 39 PICU and *n* = 50 healthy controls. The primary earner was asked to provide their occupation

^b^Data available for *n* = 45 PICU and *n* = 55 healthy controls

^c^Data available for *n* = 54 healthy controls

^d^Data available for *n* = 44 PICU and *n* = 55 healthy controls

*p < 0.01

### Basal cortisol profile waking

Table 3 outlines the raw mean basal cortisol concentrations in the two groups, adjusted for gender and age. Effect sizes differences were 0.13 for waking cortisol and CAR, 0.23 for waking +30 min and 0.36 for waking +12 h cortisol. The difference in CAR percentage increase over waking levels was 7% (31% in PICU patients; 38% in controls). The log transformations achieved acceptable normalisation values for waking and waking +30 min cortisol for both groups and for CAR in the PICU group, but normalisation was imperfectly achieved for waking 12+ hours cortisol in the PICU group (kurtosis 7.3, skewness 2.24), and for CAR (kurtosis 10.00, skewness −2.27) and waking +12 h cortisol skewness (1.60) in the control group.

**Table 3:** Aggregate basal salivary cortisol concentrations (nmol/l) for paediatric intensive care unit group and healthy control group

Time	PICU group (*n* = 47)	Healthy controls (*n* = 56)
Immediately after waking	7.00 (5.94–8.05)	7.32 (6.36–8.29)
Waking +30 min	9.00 (7.53–10.48)	10.39 (9.04–11.74)
Waking +12 h	1.32 (0.88–1.75)	1.87 (1.48–2.27)
CAR	2.01 (0.57–3.45)	3.07 (1.75–4.39)

Means (95% CI) are raw values and have been adjusted based on the effect of the covariates gender and age

*CAR* cortisol awakening response

The 2 (group) × 3 (time) mixed ANCOVA on the log transformed data indicated there was no main effect of group on basal cortisol concentrations after controlling for the effects of gender and age (*F*(1, 99) = 2.63, *p* = 0.108), nor any group × time interaction *F*(1, 87, 184.71) = 0.83, *p* = 0.431. Furthermore, the one-way ANCOVA revealed there was no main effect of group on CAR after controlling for the effects of gender and age, *F*(1, 99) = 0.56, *p* = 0.456.

To explore the influence of other possible confounders on these results, we carried out bivariate correlations in the PICU admitted group between raw cortisol values and a history of chronic illness or critical care admissions, use of steroid medication during the critical admission and taking medication at the time of the cortisol collection (for sickle cell disease, diabetes, asthma; antibiotics) but none of these were statistically significant (at *p*’s <0.05).

### Predictors of PTSD symptoms

In the PICU group, 33 of the 47 (70%) children were old enough to complete the IES-8 and had paired cortisol data for each time point. The mean (BCa 95% CI) IES-8 score was 13.12 (9.45, 17.09). The proportion scoring at risk for PTSD was 12/33 (36.4%).

The variables associated with posttraumatic stress symptoms in the univariate and multivariate analyses are shown in Table 4. Additional descriptive data for the significant univariate predictors of PTSD symptoms in the PICU group split by PTSD risk are provided in online resource 1. The univariate analysis in Table 4 revealed that the vulnerability factors, ethnicity (other) and past health problems, were associated with posttraumatic stress symptoms (*p* = 0.07 and *p* = 0.04, respectively). Of the critical illness variables, sepsis was significantly associated (*p* = 0.03) and, of the cortisol variables, evening cortisol concentration (waking +12 h) (*p* = 0.004). When included in the multivariate analysis, only past health problems and sepsis were shown to be significant independent predictors of posttraumatic stress symptoms (*p* < 0.04), with the final model accounting for 46% of the variance in scores (*p* = 0.001).

**Table 4:** Predictors of posttraumatic stress symptoms in paediatric intensive care patients (*n* = 33)

	Univariate Analysis		Multivariate Analysis				Model *R* ^2^	Model *P*
	*R* ^2^	*P*	*B*	95% CI *B*	*β*	*P*		
Impact of Events Scale Total score								
Model 1							0.21	0.03
Constant			−0.47	−12.37, 11.42				
Ethnicity^a^	0.11	0.07	6.33	−1.39, 14.05	0.28	0.10		
Past health problems^b^	0.13	0.04	9.00	−0.33, 18.33	0.32	0.06		
Model 2							0.40	0.002
Constant			−8.55	−20.34, 3.25				
Ethnicity^a^			3.80	−3.23, 10.82	0.17	0.28		
Past health problems^b^			12.97	4.32, 21.63	0.47	0.005		
Sepsis	0.14	0.03	10.99	3.71, 18.28	0.47	0.004		
Model 3							0.46	0.001
Constant			−66.98	−139.88, 5.91				
Ethnicity^a^			3.84	−2.99, 10.67	0.17	0.26		
Past health problems^b^			10.61	1.71, 19.52	0.38	0.02		
Sepsis			8.36	0.56, 16.15	0.36	0.04		
Cortisol (Waking +12 h)	0.24	0.004	61.34	−14.22, 136.89	0.26	0.11		

*B* unstandardized coefficient, *β* (beta) standardised coefficient

^a^White versus all other categories: a positive regression coefficient means worse outcome for those of a non-white ethnicity

^b^Good health versus intermediate/poor health in the six months prior to PICU admission: a positive regression coefficient means worse outcome for those with past health problems

## Discussion

The results of this exploratory study of basal cortisol regulation in 5- to 16-year-old survivors of critical illness show comparable diurnal cortisol values to matched healthy controls’ at 3–6 month follow-up. In the older study group (over 8 years), the study documents associations between higher evening cortisol levels and PTSD symptoms, but from multivariate analysis the predictive power of evening cortisol for PTSD symptoms was modest and modulated by septic illness and pre-morbid health problems. The study is, however, exploratory and requires confirmation and replication in other samples.

### Cortisol levels in PICU survivors and in controls

Admission to paediatric intensive care was not linked to significant perturbations to the HPA axis as measured by diurnal salivary cortisol concentrations at follow-up. Whilst previous research has shown alterations of circadian cortisol rhythms during critical care admissions [6, 33] our study points to normalised rhythms 3–6 months later, with concentrations following established diurnal profiles of cortisol and UK child population standards [22, 34, 35] and possibly reflecting the acute nature of the stress, the intact biological stress reactivity which had favoured survival from critical illness [6, 8] and the resumption of normal daily activities. The fact that our study found no statistically significant differences between the PICU and healthy control groups could potentially be due to two reasons: lack of power in the study caused by an insufficient sample size, or a truly small non-clinically significant difference in outcome between groups. A previous study comparing people with PTSD and controls reported a difference between the groups in percentage CAR change over cortisol waking levels of 70% (100 vs 30%) [37]. This contrasts with the current study, where this difference was only 7% (38–31%). In addition, the differences in cortisol values expressed as standardised effects sizes were small. This suggests that the differences between groups were not clinically significant and that although this was an exploratory study without an a priori sample size calculation, the lack of statistical significance would appear to be primarily due to small differences in outcome between groups.

### Cortisol levels and post-traumatic stress symptoms

From univariate analysis, there were significant positive correlations between evening (12 h after waking) cortisol concentrations and PTSD symptoms. These results may be aligned with findings of high pre-bedtime cortisol levels and PTSD symptoms in children following recent psychosocial traumas [12] supporting the notion that comparable biological mechanisms may be involved following psychological and acute physical stressors, and that anomalies in children’s ability to regulate cortisol levels in response to everyday events may possibly contribute to their stress symptoms. High evening cortisol levels may indicate overdrive or overactivity of the biological stress response which becomes manifest with exposure to daily stresses and events over the course of the day, possibly contributing to the development of PTSD. We cannot, however, exclude the possibility that PTSD symptoms themselves bring about an added psychological stress to children’s everyday lives and drive the increase in evening cortisol levels. It is moreover also possible that cortisol dysregulation and a predisposition to develop PTSD symptoms are already present in some children prior to the critical illness.

From multivariate analysis evening cortisol levels failed to independently and significantly predict PTSD symptoms over and above the presence of septic critical illness and a history of prior health problems, although it still contributed to a modest degree (*p* value of 0.11). Studies of children with traumatic injuries [17, 18] have reported positive associations between cortisol levels on admission and PTSD symptoms at short-term follow-up. It is possible that—as is the case for children exposed to psychosocial trauma [36]—the relation between cortisol and PTSD symptoms weakens over time following critical illness.

We have previously found links between sepsis and the subsequent development of PTSD symptoms [2] and in our current analyses, sepsis weakened the association between evening cortisol and PTSD symptoms. This could be related to the complex and bidirectional cortisol changes that take place during septic illness, whereby both high and low cortisol levels have been associated with increased illness severity and poor physical outcomes [6–9] but also possibly to the use of corticosteroid medication in the septic group, as there is some evidence of links between use of corticosteroids for septic shock and critical illness and PTSD symptoms [1, 38, 39].

Amongst child survivors of critical illness, cortisol concentrations tend to normalise within 48 h of admission, but the usual circadian rhythms are not immediately restored [6]. High cortisol levels can persist on hospital discharge from intensive care [4]. Exploration of subsequent endocrinological and PTSD changes in relation to cortisol regulation and treatment received during PICU admission for septic and other critical illness and—given the reported links between delusional-type memories and symptoms of PTSD amongst critically ill children [40]—to the presence of illness events such as delirium might be a fruitful area for future enquiry. This could help clarify whether PTSD symptoms and high evening cortisol levels at follow-up are related to variations in the cortisol response during critical illness.

### Strengths and limitations

Our study has a number of strengths. To the best of our knowledge, it is the first to test whether critical illness is linked to subsequent HPA function. We obtained specimens of cortisol over two test days, aggregating concentrations from each time-point to increase reliability [41]. Moreover, our protocol fidelity checks indicate that the compliance achieved was acceptable. The sample size of the comparison between PICU survivors and controls was comparatively large and the study used a well-matched control group. We did not examine the possibly confounding effects on the cortisol findings of prior abuse and neglect experiences, but the fact that non-intact families and a prior history of emotional and behavioural difficulties were comparatively uncommon in either PICU or control groups, together with the normative cortisol values demonstrated suggests that this was not likely to be a significant confounder. We did not have data on pubertal status, but our analyses are controlled for age and gender, which would have minimised the confounding effects of puberty.

This study, however, is exploratory and subject to limitations. First, we did not have measures of cortisol changes whilst in PICU and the concurrent assessment of salivary cortisol and psychological sequelae would prevent assertions about cause and effect. Secondly, we did not include a direct test of possibly more discriminating cortisol stress reactivity. Thirdly, our measures of psychological outcome are based on questionnaires and are indicative of psychiatric risk, not of actual psychopathology. Fourthly we failed to achieve full data normalisation for the statistical analysis. Finally the numbers in the analysis of associations between PTSD and cortisol were small and may have led to the loss of statistical significance on multivariate analysis.

## Conclusion

Our results indicate that children with critical illness of heterogeneous aetiologies may not as a group necessarily develop subsequent alterations in basal cortisol diurnal profiles. In the older study group of children over eight years, we did identify an association between evening cortisol level and PTSD symptoms 3–6 months following PICU discharge, but from multivariate analysis the strength of this association was reduced and modulated by the presence of septic illness and of pre-morbid health problems. Future work could usefully examine links between cortisol function both during and after critical illness and subsequent PTSD in these children.

## Electronic supplementary material

Below is the link to the electronic supplementary material.

Supplementary material 1 (DOCX 16 kb)

## References

[1] Davydow DS, Gifford JM, Desai SV, Needham DM, Bienvenu OJ (2008). Posttraumatic stress disorder in general intensive care unit survivors: a systematic review. Gen Hosp Psychiatry.

[2] Als L, Picouto MD, Hau SM, Nadel S, Cooper M, Pierce CM, Kramer T, Garralda ME (2015). Mental and physical well-being following admission to paediatric intensive care. Pediatr Crit Care Med.

[3] Diaz-Caneja A, Gledhill J, Weaver T, Nadel S, Garralda E (2005). A child’s admission to hospital: a qualitative study examining the experiences of parents. Intensive Care Med.

[4] Goodman S, Sprung CL, Ziegler D, Weiss YG (2005). Cortisol changes among patients with septic shock and the relationship to ICU and hospital stay. Intensive Care Med.

[5] Boonen E, Vervenne H, Meersseman P (2013). Reduced cortisol metabolism during critical illness. N Engl J Med.

[6] Joosten K, De Kleijn E, Westerterp M, De Hoog M, Eijck F, Hop W (2000). Endocrine and metabolic responses in children with meningoccocal sepsis: striking differences between survivors and nonsurvivors. J Clin Endocrinol Metab.

[7] Riordan FAI, Thomson APJ, Ratcliffe JM, Sills JA, Diver MJ, Hart CA (1999). Admission cortisol and adrenocorticotrophic hormone levels in children with meningococcal disease: evidence of adrenal insufficiency?. Crit Care Med.

[8] den Brinker M, Joosten K, Liem O, de Jong F, Hop WCJ, Hazelzet JA, van Dijk M, Hokken-Koelega ACS (2005). Adrenal insufficiency in meningococcal sepsis: bioavailable cortisol levels and impact of interleukin-6 levels and intubation with etomidate on adrenal function and mortality. J Clin Endocrinol Metab.

[9] Küçükemre Aydin B, Demirkol D, Bas F (2014). Evaluation of endocrine function in children admitted to pediatric intensive care unit. Pediatr Int.

[10] Mennon K, Clarkson C (2002). Adrenal function in pediatric critical illness. Pediatr Crit Care Med.

[11] Bauer AM, Quas JA, Boyce WT (2002). Associations between physiological reactivity and children’s behavior: advantages of a multisystem approach. J Dev Behav Pediatr.

[12] Carrion VG, Weems CF, Ray RD, Glaser B, Hess LD, Reiss AL (2002). Diurnal salivary cortisol in pediatric posttraumatic stress disorder. Biol Psychiatry.

[13] Pfeffer CR, Altemus M, Heo M, Jiang H (2007). Salivary cortisol and psychopathology in children bereaved by the September 11, 2001 terror attacks. Biol Psychiatry.

[14] Danese A, McEwen BS (2012). Adverse childhood experiences, allostasis, allostatic load, and age-related disease. Physiol Behav.

[15] Cicchetti D (2013). Review: Resilient functioning in maltreated children—past, present, and future perspectives. J Child Psychol Psyc.

[16] Hollocks MJ, Pickles A, Howlin P (2016). Dual Cognitive and Biological Correlates of Anxiety in Autism Spectrum Disorders. J Autism Dev Disord.

[17] Delahanty DL, Nugent NR, Christopher NC, Walsh M (2005). Initial urinary epinephrine and cortisol levels predict acute PTSD symptoms in child trauma victims. Psychoneuroendocrin.

[18] Pervanidou P, Kolaitis G, Charitaki S, Margeli A, Ferentinos S, Bakoula C, Lazaropoulou C, Papassotiriou I, Tsiantis J, Chrousos GP (2007). Elevated morning serum interleukin (IL)-6 or evening salivary cortisol concentrations predict posttraumatic stress disorder in children and adolescents six months after a motor vehicle accident. Psychoneuroendocrin.

[19] Rosmalen JGM, Oldehinkela AJ, Ormela J, de Wintera AF, Buitelaar JK, Verhulst FC (2005). Determinants of salivary cortisol levels in 10–12 year old children; a population-based study of individual differences. Psychoneuroendocrin.

[20] Bartels M, de Geus EJC, Kirschbaum C (2003). Heritability of daytime cortisol levels in children. Behav Genet.

[21] Als L, Nadel S, Cooper M, Pierce CM, Sahakian BJ, Garralda ME (2013). Neuropsychological function five months following admission to paediatric intensive care with meningo-encephalitis, sepsis, and other disorders: a prospective study of school-aged children. Crit Care Med.

[22] O’Connor TG, Ben Schlomo Y, Heron J, Golding J, Adams D, Glover V (2005). Prenatal anxiety predicts individual differences in cortisol in pre-adolescent children. Biol Psychiatry.

[23] Arafah BM, Nishiyama FJ, Tlaygeh H, Hejal R (2007). Measurement of salivary cortisol concentration in the assessment of adrenal function in critically ill subjects: a surrogate marker of the circulating free cortisol. J Clin Endocrinol Metab.

[24] Woodside DB, Winter K, Fisma S (1991). Salivary cortisol in children: correlations with serum values and effect of psychotropic drug administration. Can J Psychiatry.

[25] Clements AD, Parker CR (1998). The relationship between salivary cortisol concentrations in frozen versus mailed samples. Psychoneuroendocrin.

[26] Herrington CJ, Olomu IN, Geller SM (2004). Salivary cortisol as indicators of pain in preterm infants: a pilot study. Clin Nurs Res.

[27] Slater A, Shann F, Pearson G (2003). Paediatric Index of Mortality (PIM) Study Group: pIM2: a revised version of the Paediatric Index of Mortality. Intensive Care Med.

[28] Dyregrov A, Yule W *(*1995) Screening measures: the development of the UNICEF screening battery. In: Paper presented at the 9th Annual Meeting of the International Society of Stress Studies. Boston, MA

[29] Perrin S, Meiser-Stedman R, Smith P (2005). The Children’s Revised Impact of Event Scale (CRIES): validity as a screening instrument for PTSD. Behav Cogn Psychoth.

[30] Fries E, Dettenborn L, Kirschbaum C (2009). The cortisol awakening response (CAR): facts and future directions. Int J Psychophysiol.

[31] Kirschbaum C, Kudielka BM, Gaab J, Schommer NC, Hellhammer DH (1999). Impact of gender, menstrual cycle phase, and oral contraceptives on the activity of the hypothalamus-pituitary-adrenal axis. Psychosom Med.

[32] Oskis A, Loveday C, Hucklebridge F, Thorn Clow A (2009). Diurnal patterns of salivary cortisol across the adolescent period in healthy females. Psychoneuroendocrin.

[33] Bornstein SR, Licinio J, Tauchnitz R, Engelmann L, Negrao AB, Gold P, Chrousos GP (1998). Plasma leptin levels are increased in survivors of acute sepsis: associated loss of diurnal rhythm in cortisol and leptin secretion. J Clin Endocrinol Metab.

[34] Pruessner JC, Wolf OT, Hellhammer DH, Buske-Kirschbaum A, von Auer K, Jobst S, Kaspers F, Kirschbaum C (1997). Free cortisol levels after awakening: a reliable biological marker for the assessment of adrenocortical activity. Life Sci.

[35] O’Donnell KJ, Glover V, Jenkins J, Browne D, Ben-Shlomo Y, Golding J, O’Connor TG (2013). Prenatal maternal mood is associated with altered diurnal cortisol in adolescence. Psychoneuroendocrin.

[36] Weems CF, Carrion VG (2007). The association between PTSD symptoms and salivary cortisol in youth: the role of time since the trauma. J Trauma Stress.

[37] Wessaa M, Rohlederb M, Kirschbaumb C, Flora H (2006). Altered cortisol awakening response in posttraumatic stress disorder. Psychoneuroendocrin.

[38] Schelling G, Kilger E, Roozendaal B, de Quervain DJ, Briegel J, Dagge A (2004). Stress doses of hydrocortisone, traumatic memories, and symptoms of posttraumatic stress disorder in patients after cardiac surgery: a randomized study. Biol Psychiatry.

[39] Picouto MD (2012) Psychological function and biological stress markers in children following admission to intensive care. Dissertation, Universidad de Alcala, Spain

[40] Colville G, Kerry S, Pierce C (2008). Children’s factual and delusional memories of intensive care. Am J Respir Crit Care Med.

[41] Gunnar MR, Vasey MW, Dadds MR (2001). The role of glucocorticoids in anxiety disorders. A critical analysis. The developmental psychopathology of anxiety.

