# Supplementary material for: Stress hormones and posttraumatic stress symptoms following paediatric critical illness: an exploratory study
Source: Eur Child Adolesc Psychiatry. 2016 Dec 19;26(5):511–9. doi: 10.1007/s00787-016-0933-3 (PMC5394132; doi:10.1007/s00787-016-0933-3)
Supplement: Supplementary file 1 — Supplementary material 1 (DOCX 16 kb) [file 787_2016_933_MOESM1_ESM.docx]

**SUPPLEMENTARY INFORMATION: ESM_1.docx**

**Submission Title:**

Stress hormones and posttraumatic stress symptoms following paediatric critical illness: an exploratory study

**Authors:**

Lorraine C. Als^a^, Maria D. Picouto^b^, Kieran J. O’Donnell^c^, Simon Nadel^d^, Mehrengise Cooper^d^, Christine M. Pierce^e^, Tami Kramer^a^, Vivette Glover^f^, M. Elena Garralda^a^

**Corresponding author**: Professor Elena Garralda

Imperial College London, Hammersmith Hospital Campus - Email: e.garralda@imperial.ac.uk

**ESM_ 1.docx**

Descriptives for the univariate predictors of posttraumatic stress symptoms in the whole paediatric intensive care unit (PICU) group, split by the categorical variable PTSD risk.

| Significant univariate predictors of PTSD symptoms | Whole PICU group  (n = 33) | PTSD risk PICU group  (n = 12) | No PTSD risk PICU group  (n = 21) | PTSD risk vs  No PTSD risk  *P value* |
| --- | --- | --- | --- | --- |
| Ethnicity (other) | 14 (42%) | 8 (67%) | 6 (29%) | .066 |
| Past Health problems | 7 (21%) | 5 (42%) | 2 (10%) | .071 |
| Sepsis | 13 (39%) | 7 (58%) | 6 (29%) | .142 |
| Waking +12h cortisol concentration (nmol/l)^a^ | 1.45 (0.24) | 2.21 (0.52) | 1.01 (0.17) | .007 |

PTSD risk was classified as a score ≥17 on the Impact of events-8 (IES-8) Scale.

Data are frequency (percent) or mean (SEM). Comparisons made using Paired Exact Tests (for categorical data) or T-Test (for continuous data).

^a^ Means (SEM) are based on raw values; T-test was conducted on log-transformed values.
